# Pemphigus Foliaceus—Repeated Treatment With Rituximab 7 Years After Initial Response: A Case Report

**DOI:** 10.3389/fmed.2018.00315

**Published:** 2018-11-09

**Authors:** Magdalena Kraft, Margitta Worm

**Affiliations:** Department of Dermatology, Venerology and Allergology, Allergy-Center-Charité, Charité–Universitätsmedizin Berlin, Berlin, Germany

**Keywords:** pemphigus foliaceus, rituximab, anti-CD20, b-cell depletion, autoimmune blistering diseases

## Abstract

Pemphigus foliaceus is an autoimmune skin disease mediated by autoantibodies directed against desmoglein-1 located in the upper epidermal layer. Rituximab, a monoclonal anit-CD20 antibody depleting b-cells, offers an effective treatment possibility for therapy-resistant pemphigus foliaceus. Here, we present the case of 55-year-old man who did not respond sufficiently to conventional treatment with prednisolone, azathioprine, and cyclophosphamide, but underwent almost complete remission after rituximab treatment. The patient relapsed 7 years later, and a repeated course of rituximab infusions led to a partial remission.

## Introduction

Pemphigus foliaceus is a rare, autoimmune blistering skin disease with an estimated incidence of < 1 million individuals in the USA and Europe (1). It is caused by autoantibodies directed against desmoglein-1 (Dsg-1), a glycoprotein important for intercellular adhesion between keratinocytes (2, 3). The disease manifests as erythematous papules, plaques, and erosions with scaly crusts. The involvement of seborrheic skin areas is typical for the disease. The loss of adhesion occurs in the upper epidermal layers and is limited to the skin with no mucosal involvement; this is attributable to the expression pattern of Dsg-1 and distinguishes pemphigus foliaceus from the more common pemphigus vulgaris (4, 5).

Glucocorticoids are the first-line treatment for pemphigus foliaceus. Treatment with other immunosuppressants such as azathioprine, mycophenolate mofetil or methotrexate, is also well-established (6). Recent data suggest that a b-cell-depleting therapy with rituximab is highly effective in treating pemphigus vulgaris, but also pemphigus foliaceus (7–10). Here, we describe the case of a patient suffering from a therapy-resistant pemphigus foliaceus; the patient was in remission for 7 years after initial rituximab treatment and responded well to the repeated treatment.

## Case presentation

**Figure 1:**
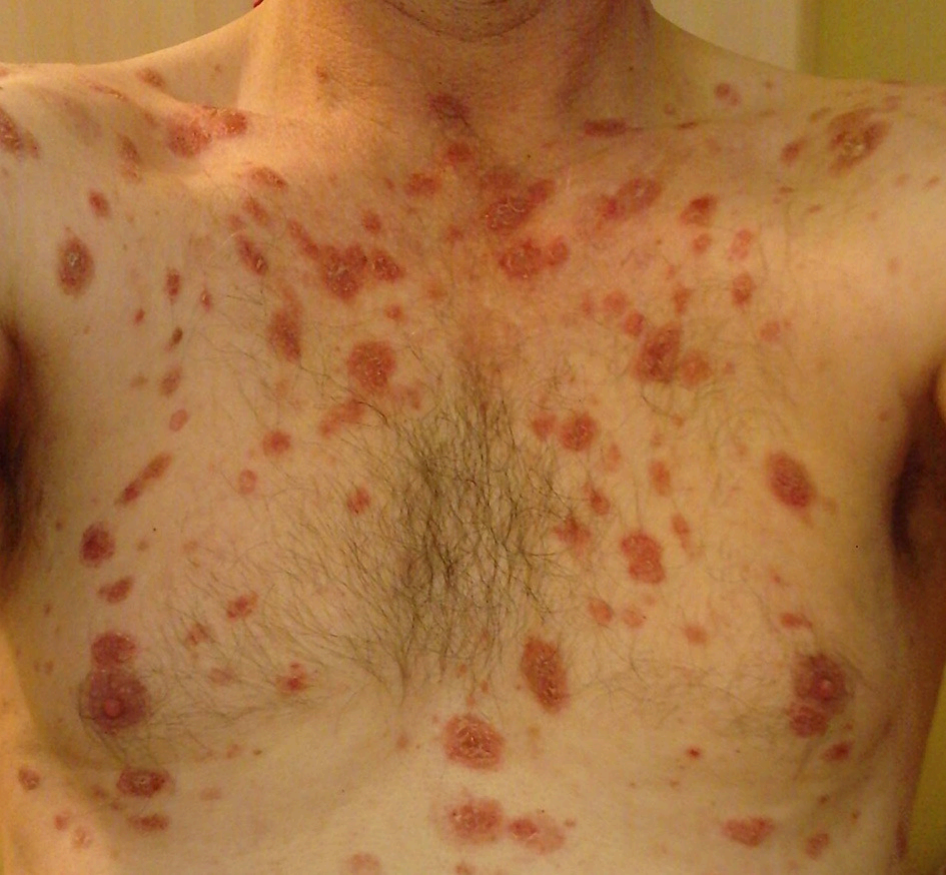
Skin lesions before rituximab treatment.

A 55-year-old man with a history of progressing skin lesions over the past 8 months visited our department for the first time in spring 2011. The clinical examination revealed multiple erythematous papules and plaques with crusts on his back, chest, face, and scalp (about 40% of body surface area was involved) with no mucosal involvement (Figure 1). The patient presented no other symptoms and had no chronic diseases or allergies. His blood tests revealed a highly elevated Dsg1 antibody level (130 U/ml; normal range < 20 U/ml) and a slightly elevated γ-glutamyltransferase level. Differential blood count, liver enzymes, creatinine, and Dsg3 antibody level were within the normal range. Histological examination of the patient's skin biopsy revealed an inflammatory infiltrate, eosinophilic spongiosis, and superficial epidermal blister formation.

**Figure 2:**
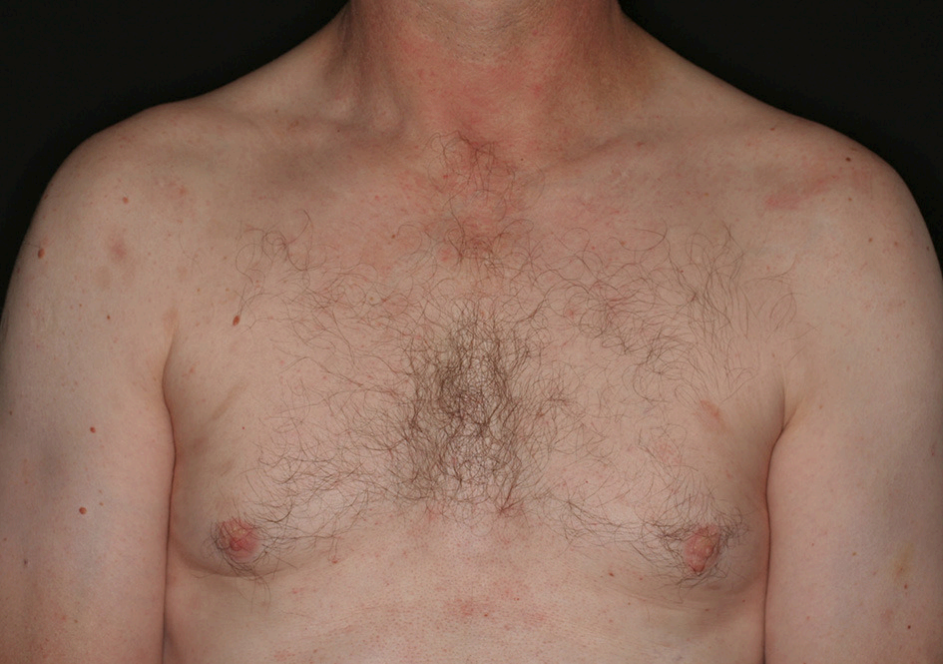
Clinical picture eight months after initial rituximab treatment.

Based on the findings, pemphigus foliaceus was diagnosed and a treatment with prednisolone (10 mg/day) and azathioprine (100 mg/day) was started. Topical therapy with clobetasol propionate and chlorhexidine was also initiated. Furthermore, methylprednisolone infusions (750 mg) were administered once a month for 3 months. This treatment did not result in complete remission; thus, methylprednisolone was replaced with dexamethasone (300 mg) and cyclophosphamide infusions (500 mg) once a month. Azathioprine had to be discontinued due to increasing liver enzymes. The treatment with cyclophosphamide and glucocorticoids was discontinued after 5 months without achieving remission. Hence, we next treated the patient with rituximab. Therefore, two rituximab infusions (1 g each) were administered 2 weeks apart leading to a near-complete b-cell depletion in peripheral blood, a decrease in Dsg1 antibody levels (below the detection range), and an almost complete remission of the skin lesions within the next year (Figure 2). Consecutively, therapy with prednisolone (10 mg/day) and topical mometasone furoate was continued and in the following 2 years, the prednisolone dose was reduced to 5 mg/day. The patient remained in remission for 7 years with this therapy (with Dsg1 antibody levels continuously within the normal range). However, in autumn 2017, skin lesions reappeared, which was accompanied by an increase in the Dsg1 antibody levels (75 U/ml). The prednisolone dosage was increased (temporarily up to 60 mg/day), but it was not sufficient to control the disease. Therefore, rituximab infusions (2 × 1 g within 14 days) were readministered, which led to slow continuous healing of the skin lesions.

## Discussion

Numerous case reports and studies have reported on the efficacy of rituximab in treating pemphigus vulgaris but also pemphigus foliaceus (1, 7–11). Recently, Joly et al. (10) published a randomized, multi-center, open-labeled clinical study comparing rituximab and prednisolone as the first line of treatment for pemphigus. The study subjects were treated with 1,000 mg rituximab on days 0 and 14, followed by 500 mg rituximab after 12 and 18 months and 0.5–1 mg/kg prednisolone tapered over 3–6 months. The control group was treated with 1–1.5 mg/kg prednisolone tapered over 12–18 months. After 2 years, the achieved remission rates were 89 and 34% for the rituximab and the control group, respectively. In the control group, the rate of serious adverse events was twice as high as that among the rituximab-treated group.

Thus, rituximab is effective in treating pemphigus foliaceus; however, its ideal dosage regimen is still unknown. In most cases, either 375 mg/m^2^ rituximab is administered once a week for 4 weeks (lymphoma protocol) or two doses of 1,000 mg rituximab are administered 2 weeks apart (rheumatoid arthritis protocol). In their comprehensive review, Ahmed and Shetty (11) reported that responder rates (in pemphigus vulgaris) for both protocols were similar. Because of the low B-cell burden in autoimmune diseases, some authors proposed a low dosage regimen (12–14).

Relapse of pemphigus after rituximab treatment is common (15). In majority of the patients, remission can be maintained up to a few years. A 7-year remission duration, as observed in the current study, is not frequent. Joly et al. reported that 25% of rituximab treated patients relapsed within 2 years (10). Colliou et al. followed 21 rituximab treated patients for 7 years (16). The relapse occurred in 81% of patients and was associated with either persisting or, as in our case, reincreasing Dsg antibody levels. The factors influencing the remission time are not well-characterized (17). The associations between prolonged time to relapse and either higher number of rituximab cycles or a high-dose regiment were described (7, 15, 17, 18). On the other hand, prophylactic administration of an additional rituximab infusion in patients in remission was shown not to be beneficial (19). Saleh (20) reported a correlation between Dsg1 antibody levels at baseline and time to relapse: patients with high levels of Dsg1 antibodies relapsed usually within one year after rituximab treatment, while patients with low Dsg1 antibody levels stayed in remission for about 2 years. Interestingly there was no association between time to relapse and Dsg3 antibody levels or clinical severity at the time of rituximab treatment.

In a recent small study, Keeley et al. reported that a low-dose maintenance immunomodulatory treatment after rituximab therapy might prevent a relapse (21). Ahmed et al. observed no relapses in ten patients treated with immunoglobulins and rituximab (22). The adjuvant immunoglobulins were administered in average for 34 months following the rituximab treatment and a long-lasting remission was achieved in all patients [average follow up was 7 years after the initial rituximab treatment (22)]. In our patient, it is probable that the prolonged remission was supported by the continuous low-dose prednisolone therapy.

As here observed, treatment with rituximab is successful in the majority of the patients after relapse (15, 18, 23) so that repeated treatment can be recommended for these patients.

Serious adverse event following rituximab treatment are rare. Those are mostly infections (e.g., viral hepatitis reactivation, or herpes virus infections) (24). Few cases of infections with lethal outcomes after rituximab treatment have been reported (24); thus, patients and physicians should be aware of this risk and immediately act in case of suspected adverse event.

## Concluding remarks

Our case report demonstrates that rituximab can be effective in treating therapy-resistant pemphigus and long-lasting remission may be achieved. A low-dose maintenance immunomodulatory treatment after rituximab therapy may prolong the remission stage. In case of a relapse, repeated treatment with rituximab is usually successful. However, this therapy is limited due to the high cost of rituximab and the risk of rare but severe side effects.

## Author contributions

MK wrote the manuscript. MW contributed to conception of the manuscript and revised it critically.

### Conflict of interest statement

The authors declare that the research was conducted in the absence of any commercial or financial relationships that could be construed as a potential conflict of interest.
